# High-Humidity Incubation Improves Post-Microinjection Viability and Larval Performance of CRISPR/Cas9-Edited *Helicoverpa armigera* Embryos

**DOI:** 10.3390/insects16121257

**Published:** 2025-12-11

**Authors:** Jingyun Zhu, Hongran Li, Yan Peng, Minghui Jin, Kaikai Mao, Yutao Xiao

**Affiliations:** 1 Shenzhen Branch, Guangdong Laboratory of Lingnan Modern Agriculture, Key Laboratory of Gene Editing Technologies (Hainan), Ministry of Agriculture and Rural Affairs, Agricultural Genomics Institute at Shenzhen, Chinese Academy of Agricultural Sciences, Shenzhen 518000, China; jingyun_zhu@126.com (J.Z.); lihongran@caas.cn (H.L.); pengyan@caas.cn (Y.P.); jinminghui@caas.cn (M.J.); 2 Key Laboratory of Integrated Pest Management on Tropical Crops of the Ministry of Agriculture and Rural Affairs, Environment and Plant Protection Institute, Chinese Academy of Tropical Agricultural Sciences, Haikou 571101, China; 3 Guangxi Key Laboratory of Agro-Environment and Agric-Products Safety, College of Agriculture, Guangxi University, Nanning 530004, China; kaikaimao@gxu.edu.cn

**Keywords:** CRISPR/Cas9, high-humidity incubation, *Helicoverpa armigera*

## Abstract

Gene editing technologies are playing an increasingly important role in the study of insect gene functions. Appropriate incubation humidity helps improve the hatching rate of insect eggs. However, to date, no study has evaluated the impact of humidity on the hatching rate of gene-edited eggs or the subsequent development of the larvae. Using the cotton bollworm *Helicoverpa armigera* (Lepidoptera: Noctuidae) as a model, we investigated the weight and hatching rate of microinjected eggs under different humidity conditions. We determined the larval development time and pupal weight and calculated the larval gene editing efficiency. The results showed that post-microinjection incubation under high humidity (RH = 95%) had a significant influence on CRISPR/Cas9 gene-edited eggs. The key research results indicate that, compared with the incubation environment with RH ≤ 80%, after 48 h of incubation under 95% RH, the hatching rate of *H. armigera* eggs increased by more than 27.5%, with higher egg saturation and greater egg weight; meanwhile, the larval developmental duration was shorter and the pupal weight was also greater. These results establish a humidity-regulated developmental recovery protocol for gene-edited insects, which provides theoretical and practical support for optimizing post-microinjection recovery processes in gene-edited insects.

## Simple Summary

This study focused on solving a key gap in insect gene editing: while humidity affects normal insect egg hatching, its role in post-microinjection recovery of CRISPR/Cas9-edited *Helicoverpa armigera* (cotton bollworm) eggs remained unstudied. Lab-bred *H. armigera* eggs (laid within 2 h) were injected with CRISPR/Cas9 (targeting the *TRPA1* gene) or a control solution, then incubated at 50%, 65%, 80%, or 95% relative humidity (RH) for 48 h. Results showed 95% RH was optimal: eggs stayed plump (avoiding shriveling at lower RH), with 100-egg weight peaking at ~103 mg and hatching rate rising over 27.5% vs. RH ≤ 80%. Larvae from these eggs also developed faster and had heavier pupae, while gene editing efficiency remained unchanged across humidities. This 48-h 95% RH incubation protocol boosts post-injection viability of *H. armigera* eggs, offering practical support for efficient insect gene editing and a reference for other species.

## 1. Introduction

With the advancement of gene sequencing technologies, gene editing systems have emerged as a powerful tool in biological sciences and an essential approach for insect gene function studies [1,2,3,4]. Insect eggs often require specific pretreatments before editing, which typically vary between species. For species with soft eggs, freshly collected eggs can be placed directly onto double-sided adhesive tape on a microscope slide and injected. Eggs with a soft chorion but high internal pressure should be dried before injection, while thick-shelled eggs should be softened and dried prior to injection [5]. The timing of the injection of eggs is also crucial, and to reduce the occurrence of chimeras, most experiments require this operation to be performed within 2 h after egg laying. Studies have shown that the earlier the injection is performed, the higher the deletion frequency [3,6].

Environmental humidity plays a critical role in insects’ growth and development, influencing their egg hatch, developmental rate, fecundity, and body coloration [7,8,9,10,11]. For *Halyomorpha halys*, low humidity reduces the survival rate of first-instar nymphs but exerts no effect on third- to fourth-instar nymphs. In contrast, high humidity enhances the survival rate of first-instar nymphs while decreasing that of third- to fourth-instar nymphs [7]. Low environmental humidity delays egg development in *Callosobruchus maculatus* [9], yet it does not have this effect on *Halyomorpha halys* [7]. Low-humidity environments in *Picromerus lewisi* can not only prolong the developmental duration of eggs and reduce their hatching rates but also shorten the adult lifespan [10]. Notably, distinct species—and even different immature stages of the same species—exhibit specific humidity tolerance ranges [12]. Maternal oviposition behaviors—such as selecting optimal laying sites and clustering eggs—play a critical role in protecting eggs against desiccation [13]. Additionally, egg survival can be enhanced when mothers provide adequate internal water reserves, increase hydrocarbon content in the eggshell [14], or coat the eggs with substances such as hydrogels [15,16,17,18]. The deposited egg is also capable of protecting itself against desiccation, the serosa and its cuticle make a significant contribution to the egg’s drought resistance [19,20]. Notably, in *Aedes*, *Anopheles*, and *Culex* mosquitoes, the formation of dark eumelanin in the endochorion may further enhance the serosa’s contribution to drought resistance [21]. Diapause also enhances drought resistance in *Aedes aegypti* eggs [22].

In most cases, eggs utilized for genetic editing are relatively fragile due to being laid at an early developmental stage prior to full maturation. The pretreatment and the injection process of eggs can also cause certain damage to the eggs. It is therefore imperative to enhance the hatching rate of eggs post injection. This study focuses on *H. armigera* and investigates the effects of different humidity conditions on the recovery process of eggs following microinjection. The results showed that early-stage humidity incubation not only had a significant impact on the weight of *H. armigera* eggs after microinjection but also affected the hatching rate, larval developmental duration, and pupal weight.

## 2. Materials and Methods

### 2.1. Insect Rearing

The cotton bollworm (*Helicoverpa armigera*) was collected from Xiajin, Shandong Province, China, in 2023. Adults trapped in the wild are paired individually. After multiple generations of breeding, the eggs laid by the offspring of these paired adults are used for experiments. Insects were reared under controlled laboratory conditions at 27 ± 1 °C, 75 ± 10% relative humidity, and a photoperiod of 14 h of light and 10 h of dark. Adult moths were provided with a 10% sucrose solution as an energy source.

### 2.2. sgRNA Design and Synthesis

A single sgRNA against *TRPA1* (Accession Number: XM_049838796) was designed using the sgRNAcas9 design tool (Table 1) (developed by independent researchers; available at BiooTools: http://www.biootools.com/, Nanjing, China) [23]. The sgRNA target sequences were checked in a search of the *H. armigera* genome using the sgRNAcas9 design tool, and no potential off-target sites were identified. A PCR-based approach was used to synthesize sgRNAs following the manufacturer’s instructions (GeneArt Precision gRNA Synthesis Kit, Thermo Fisher Scientific, Waltham, MA, USA). The Cas9 protein GeneArt Platinum Cas9 Nuclease) was purchased from Thermo Fisher Scientific (Shanghai, China). 

### 2.3. Microinjection of Embryos

Freshly laid eggs (within 2 h) were washed from gauze using 1% (*v*/*v*) sodium hypochlorite solution and rinsed with distilled water. The eggs were then stuck to a microscope slide with double-sided adhesive tape. One hundred eggs were affixed to each microscope slide.

About 2 nL of a mixture of sgRNA (300 ng/μL) and Cas9 protein (50 ng/μL) was injected into individual eggs using the CRISPR/Cas9 genome editing system as previously reported [24]. The Cas9 protein (GeneArt Platinum Cas9 Nuclease) was purchased from Thermo Fisher Scientific, mixture of sgRNAs and Cas9 protein was injected into individual eggs using the NanojectIII (Drummond Scientific, Broomall, PA, USA). Injected eggs were incubated in environments with a temperature of 25 ± 1 °C and separate humidity levels of 50%, 65%, 80%, and 95% for 48 h. After 48 h, the eggs were transferred to an environment with a temperature of 25 ± 1 °C and 80% humidity for further incubation. Temperature was maintained at 25 °C with relative humidity (RH) levels of 50%, 65%, 80%, and 95%, controlled by saturated salt solutions of NH_4_NO_3_-NaNO_3_, NaNO_2_, (NH_4_)_2_SO_4_, and Pb(NO_3_)_2_, respectively, following the method described by Winston [25].

Eggs that were injected with a mixture of H_2_O + Cas9 protein and non-injected eggs were treated as described above, serving as the experimental and control groups, respectively.

### 2.4. Measurement of Hatching Rate and 100-Egg Weight Under Experimental Conditions

Before egg attachment in Section 2.2, the total weight of each microscope slide and its double-sided tape was measured and recorded as G0. After attaching the eggs, the weight of the slide with the eggs was measured and recorded as G1. The initial weight of 100 eggs was calculated as G1 minus G0. After placing the slides in different humidity environments for 24 h, the weight of each slide was measured again and recorded as G3. The weight of the eggs after 24 h was calculated as G3 minus G0. The egg weight after 48 h was calculated using the same method. Each humidity condition was repeated four times.

The number of hatched eggs on each slide under different humidity conditions was recorded for subsequent experimental analysis. The hatching rate of the eggs is equal to the number of hatched larvae on each slide divided by 100, then multiplied by 100 (i.e., expressed as a percentage).

### 2.5. Observation and Measurement of Developmental Duration, Pupal Weight, and Pupation Rate

The hatched larvae described in Section 2.3 were individually transferred to artificial diets and reared under controlled conditions (27 ± 1 °C, 75 ± 10% relative humidity). Upon reaching the 2nd instar, 10 larvae per replicate were randomly selected for individual genomic DNA extraction and genotyping (Section 2.6). The remaining larvae were reared continuously: the number of successfully pupated individuals was recorded, and the developmental duration and pupal weight were measured.

### 2.6. Determination of Gene Editing Efficiency in Hatched Larvae

Primers used for mutation detecting were designed using Primer Premier 5 (Premier Biosoft International, Palo Alto, CA, USA) according to the sgRNA locations (Table 2). Genomic DNA was extracted with a Multisource Genomic DNA Miniprep Kit (Axygen, Corning Incorporated, Union City, CA, USA). PCR amplicons were analyzed via agarose gel electrophoresis. PCR products were recovered and cloned into T3 Vector and sequenced by Sangon Biotech (Shanghai, China).

### 2.7. Data Analysis

A statistical comparison of the hundred-egg weight and egg hatching rate across different treatment groups was performed using Student’s *t*-test. A one-way analysis of variance (ANOVA) was used to analyze differences among the hatching rate, 100-egg weight, pupation rate, pupation time, and pupal weight of *H. armigera* followed by separation of means using Fisher’s protected least significant difference (LSD) test at *p* = 0.05. The experimental data were analyzed using the package IBM SPSS Statistics 22.0 (SPSS, Inc., Chicago, IL, USA).

## 3. Results

### 3.1. Hatching Rate and 100-Egg Weight

**Figure 1:**
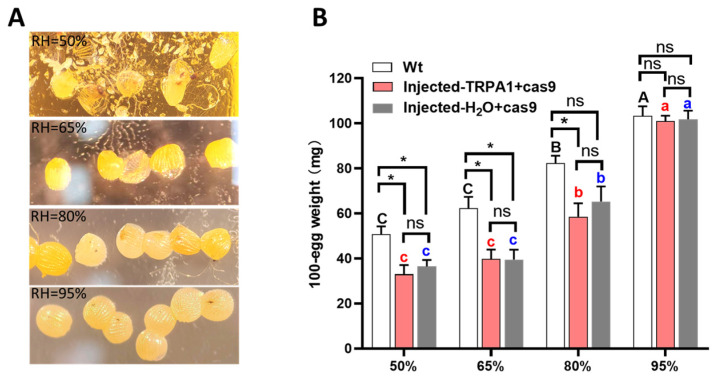
Egg weight of *H. armigera* after microinjection under different humidity incubation conditions: (**A**) *H. armigera* eggs injected with *TRPA1* + Cas9 after 48 h of incubation under different humidity levels; (**B**) weight per 100 eggs after 48 h of incubation under different humidity levels. Each humidity condition was replicated four times. Different letters in the figure indicate significant differences at *p* < 0.05 (one-way ANOVA). * represents values that were statistically significant at *p* < 0.05; ns indicates no significant difference at *p* = 0.05. The mean amounts are presented with the standard error of the mean (±SE).

At 95% relative humidity, eggs remained plump and intact, while eggs incubated under other humidity conditions showed varying degrees of shriveling (Figure 1A). Incubation under different humidity conditions had a significant effect on egg weight (Wt, F = 49.373, df = 3, 12, *p* < 0.0001; Injected-*TRPA1* + cas9, F = 31.425, df = 3, 12, *p* < 0.0001; Injected-H_2_O + cas9, F = 42.026, df = 3, 12, *p* < 0.0001). When the humidity was 95%, the weight of 100 eggs was the highest, reaching 103.25 mg, 101.00 mg, and 101.75 mg, respectively. At 50% humidity, the weight of 100 eggs was the lowest, measuring 50.75 mg, 33.00 mg, and 36.50 mg, respectively (Figure 1B). Under the same incubation conditions, there was no significant difference in egg weight between eggs injected with *TRPA1* + Cas9 and those injected with water + Cas9 (50%RH, *p* = 0.508; 65%RH, *p* = 0.969; 80%RH, *p* = 0.480; 95%RH, *p* = 0.874). In the 50% and 60% humidity environments, the egg weight of non-injected eggs was significantly higher than that of injected eggs (Wt vs. Injected-*TRPA1* + cas9: 50%RH, *p* = 0.016; 65%RH, *p* = 0.014; Wt vs. Injected-H2O + cas9: 50%RH, *p* = 0.020; 65%RH, *p* = 0.015).

**Figure 2:**
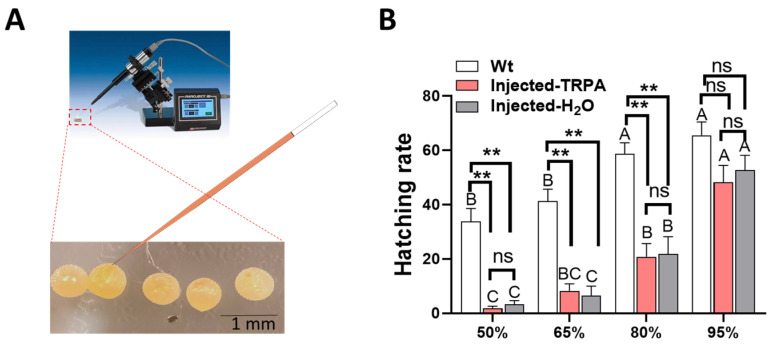
Hatching rate of *H. armigera* eggs after microinjection under different humidity incubation conditions: (**A**) diagram of the microinjection procedure; (**B**) hatching rates of eggs under different humidity conditions. Each humidity condition was replicated four times. Different letters in the figure indicate significant differences at *p* < 0.05 (one-way ANOVA). ** represents values that were statistically significant at *p* < 0.01; ns indicates no significant difference at *p* = 0.05. The mean amounts are presented with the standard error of the mean (±SE).

Different incubation humidity conditions had a significant effect on the hatching rate of *H. armigera* eggs (Wt, F = 10.359, df = 3, 12, *p* = 0.001; Injected-*TRPA1* + cas9, F = 24.561, df = 3, 12, *p* < 0.0001; Injected-H2O + cas9, F = 24.270, df = 3, 12, *p* < 0.0001; Figure 2B). When the incubation humidity was 95%, the hatching rate of *H. armigera* eggs was the highest, Wt reaching 65.50%, Injected-*TRPA1* + cas9 reaching 48.25%, and Injected-H_2_O + cas9 reaching 52.75%. There was no significant difference in the hatching rate of *H. armigera* eggs injected with *TRPA1* + Cas9 or water + Cas9 among the four humidity environments (50%RH, *p* = 0.508; 65%RH, *p* = 0.969; 80%RH, *p* = 0.480; 95%RH, *p* = 0.874; Figure 2B). The hatching rate of non-injected eggs after incubation at 50%, 65%, and 80% humidity was significantly higher than that of *TRPA1* + Cas9-injected (50%RH, *p* = 0.006; 65%RH, *p* = 0.001; 80%RH, *p* = 0.001) and H_2_O + Cas9-injected (50%RH, *p* = 0.006; 65%RH, *p* = 0.001; 80%RH, *p* = 0.003; Figure 2B) eggs.

### 3.2. Developmental Duration, Pupal Weight, and Pupation Rate

**Figure 3:**
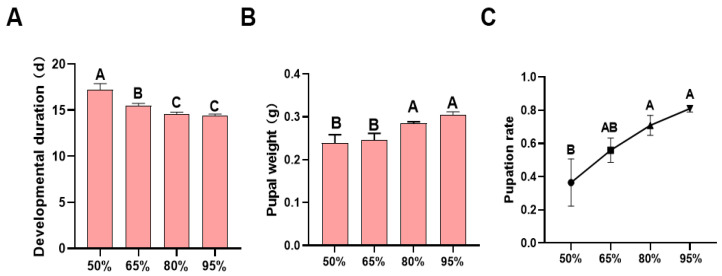
Larval performance of *H. armigera* larvae hatched after 48 h of incubation under different humidity conditions: (**A**) developmental duration; (**B**) pupal weight; (**C**) pupation rate. Different letters in the figure indicate significant differences at *p* < 0.05 (one-way ANOVA).

Incubation under different humidity conditions significantly affected the developmental duration (F = 13.636, df = 3, 96, *p* < 0.0001; Figure 3A), pupal weight (F = 10.290, df = 3, 12, *p* < 0.001; Figure 3B), and pupation rate (F = 5.033, df = 3, 12, *p* = 0.017; Figure 3C) of *H. armigera*.

### 3.3. Gene Editing Efficiency in Hatched Larvae

**Figure 4:**
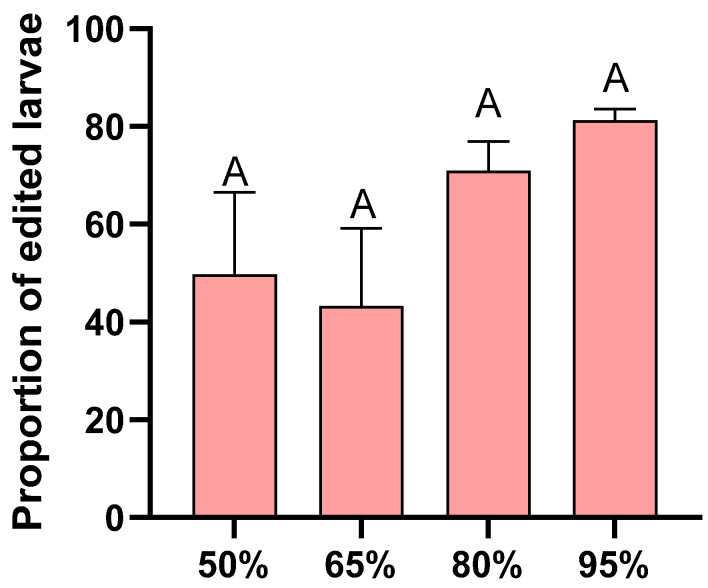
Proportion of successfully edited larvae under different humidity conditions. Each humidity condition was replicated four times. Different letters in the figure indicate significant differences at *p* < 0.05 (one-way ANOVA).

There was no significant difference in the gene editing rate between eggs incubated under different humidity conditions (F = 1.778, df = 3, 12, *p* = 0.205; Figure 4).

## 4. Discussion

Our experimental results show that the hundred-egg weight of *H. armigera* significantly decreases with decreasing humidity after microinjection (Figure 1). In a 50% humidity environment, the plumpness of *H. armigera* eggs decreases (Figure 1A), and the weight of 100 eggs is significantly lower than that in a 95% humidity environment (Figure 1B). This may be caused by a variety of factors. Bodily secretions, feces, hairs, and scales collectively form multiple defensive barriers on the egg surface [16,17,26]. These surface-based protective substances, however, are susceptible to damage by egg washing and egg disinfection. The serosa, an additional embryonic membrane that encloses the embryo and yolk and can protect the embryo from desiccation [19], can also be damaged by egg injection. The eggshell may become thinner during the pro-injection preparation process, reducing the egg’s ability to retain water. Additionally, eggs may absorb water during the washing process; some species can even obtain sufficient water from the moisture of the air [27], and this absorbed water can be lost in a dry environment.

Humidity plays a critical role in the hatching rate of insect eggs [20,28,29,30]. Eggs maintained under excessively arid conditions may fail to hatch: in some instances, this is attributable to desiccation of the internal embryo; in other cases, it arises because the chorion itself hardens to a degree that precludes emergence of the immature insect [31]. Relative humidity also can affect egg hatching time; studies on blow flies have shown that higher relative humidity leads to shorter hatching durations [28]. High-humidity environments have varying effects on the hatching of eggs from different insect species. Studies on *Spodoptera exigua* have shown that higher humidity leads to a higher egg hatching rate [30]. In contrast, research on *Hypera postica* has indicated that excessively high humidity can reduce the hatching rate of its eggs [32]. We only investigated the impact of incubation in different humidity environments for 48 h on *H. armigera* eggs after microinjection, with the gene editing targeting a single locus (TRPA1). Our results showed that different humidity incubation conditions had a significant effect on the hatching rate of *H. armigera* eggs after gene editing; the higher the humidity, the higher the hatching rate (Figure 2B). Humidity plays a critical role in the recovery process of injected eggs. Compared to the control group, both the *TRPA1* + Cas9-injected and H_2_O + Cas9-injected eggs exhibited significant reductions in both hundred-egg weight and hatching rate under low-humidity conditions. Eggs subjected to microinjection are more fragile and may therefore require higher humidity levels for successful development. This is consistent with the observed reduction in egg weight under low-humidity conditions. Our results are the first to demonstrate that humidity affects the hatching rate of eggs after microinjection targeting *TRPA1* in *H. armigera*.

Humidity influences larval development, and insects at different developmental stages exhibit varying humidity preferences. In *Halyomorpha halys*, early-instar nymphs are sensitive to drought, while later-instar nymphs are intolerant of high humidity [33]. Our study showed that early-stage humidity incubation had a significant impact on the growth and development of *H. armigera* larvae (Figure 3). The extended developmental period and decreased pupal weight may result from excessive desiccation of eggs incubated under low humidity, as varying degrees of water loss were observed at 80%, 65%, and 50% relative humidity (Figure 1). Due to their underdeveloped protective structures, early-stage eggs are particularly vulnerable to environmental stressors [34,35].

Many studies incubate insect eggs after injection in environments with approximately 70 ± 10% humidity [36,37]. Our research results indicate that in a humid environment below 80%, a certain proportion of *H. armigera* eggs can still hatch; however, the hatching rate is relatively low. To obtain gene knockout strains, it is necessary to increase the number of microinjected eggs, which will inevitably increase the usage of eggs and the workload. This study provides a strategy to improve the hatching rate of eggs after microinjection and enhance larval performance: by incubating the injected eggs under high humidity for two days. It should be noted that this strategy is currently based on experiments targeting the *TRPA1* locus in *H. armigera*, which limits the direct generalizability of our conclusions to other genes or insect taxa. Additionally, in the gene editing efficiency analysis, we genotyped 40 larvae per humidity condition but only counted edited and unedited individuals without distinguishing the presence of chimeras. This may compromise the accuracy of editing efficiency evaluation, and future studies should adopt more advanced methods (e.g., deep sequencing) to quantitatively analyze the chimerism level.

## 5. Conclusions

Our research results indicate that, compared with incubation in a low-humidity environment, the hatching rate and egg weight of *H. armigera* eggs incubated in an environment with humidity greater than 80% for 48 h after microinjection are significantly higher. Without affecting editing efficiency, the larval developmental duration is shorter, and the pupal weight is greater. Our research findings provide guidance for the efficient editing of *H. armigera* eggs and may also serve as a preliminary reference for the incubation of edited eggs of other insect species, pending further verification to confirm whether these results can be broadly applicable across different genes and taxa.

## Figures and Tables

**Table 1:** sgRNA sequence.

Primer ID	Sequence
sgRNA-*TRPA1*	GGTGCCACAGCGCACGGTCCG

**Table 2:** Primers used for mutation detecting.

Primer ID	Sequence
D-*TRPA1*-F	ATGGGTGCATCGCTAGAG
D-*TRPA1*-R	AGGAAGCGAGGACTTGCG

## Data Availability

The original contributions presented in this study are included in the article. Further inquiries can be directed to the corresponding author.
